# Demands on a continuing education online-study program for physicians

**DOI:** 10.1186/cc13200

**Published:** 2014-03-17

**Authors:** J Seifried, V Titschen, J Guttmann, S Schumann

**Affiliations:** 1 University Medical Center Freiburg, Germany

## Introduction

Physicians have an intense and irregular workday life. Thus, they need to use their time for continuing education in the most efficient way. This presents a major challenge to suppliers of continuing study programs. Our online master program Physico-Technical Medicine (PTM) addresses working physicians who want to acquire knowledge in the fields of biomedical engineering and medical physics [1]. We attach great importance to the special needs of our participants. Therefore, we investigated which general conditions are most important for the working physician regarding qualifying continuing education.

## Methods

The general conditions of our continuing education program (PTM) were examined on the basis of evaluation questionnaires. Additionally, students were asked in interviews about their demands, and they could give anonymous feedback on feedback cards.

## Results

*Flexibility*:students appreciated flexibility regarding time and environment of their learning activities. This flexibility enables the students to use even small time frames or traveling times for learning. Students can choose their preferred time and place for their learning activities. *Security in planning*: students have to organize their time frames for learning besides their daily work, their family life, and possibly their academic career. Especially phases of attendance should be planned and communicated as early as possible. *Transparency of structure*: participants ask for transparency in terms of the expected workload and expenditure of time to plan their studies and get focused. *Relation to previous knowledge*: learning success is supported by relating new contents to previous knowledge from the student's first medical degree. Mainly clinical examples illustrate abstract topics, which also helps students to recognize the relevance of the content. *Individual support*: students need contact persons in terms of learning organization, technical support, and for questions regarding the course material.

## Conclusion

We could show that an extra-occupational continuing study program for physicians should be adjusted to their special situation and therefore has to comply with particular requirements. As such we could identify five main requirements which are continuously fulfilled by the online master program PTM and regularly surveyed by evaluations.
